# Does categorizing scale scores with cutoff points affect hypothesis-testing results?

**DOI:** 10.1007/s44192-024-00067-4

**Published:** 2024-04-22

**Authors:** Ugurcan Sayili, Esin Siddikoglu, Deniz Turgut, Hamza Emre Arisli, Betul Ceyhan, Mehmet Guven Gunver, Sevda Ozel Yildiz, Eray Yurtseven, Ethem Erginoz

**Affiliations:** 1 Department of Biostatistics, Institute of Graduate Studies in Health Sciences, Istanbul University, Istanbul, Türkiye; 2 Department of Public Health, Cerrahpasa Faculty of Medicine, Istanbul University-Cerrahpasa, Istanbul, Türkiye; 3 Department of Biostatistics, Istanbul Faculty of Medicine, Istanbul University, Istanbul, Türkiye

**Keywords:** Cutoff, Scale, Sensitivity, Type 1 error, False significance

## Abstract

**Background:**

The aim of this study is to evaluate the hypothesis test results after categorizing the scale scores with cut-off points and to assess whether similar results would be obtained in that best represent the categories.

**Methods:**

This cross-sectional study was conducted between March 15 and 20, 2023 via the Lime Survey. The questionnaire included questions about the sociodemographic and life characteristics of the participants and the Beck Depression Inventory II (BDI-II). Four groups (minimal, mild, moderate, severe depression) were formed using the cutoff points. Data analysis was performed with all participants and referred to as the conventional analysis group. Then, six subanalysis groups were determined to best represent the groups formed according to the BDI-II. In each BDI-II category, six subanalysis groups were created, including those between Q1–Q3 (IQR group), including those within ± 1 std, including those between 5p–95p (90% of the sample), including those between 2.5p–97.5p (95% of the sample). In addition, 100 different samples were randomly selected containing 50% of each group.

**Results:**

Of the 1950 participants, 84.7% (n = 1652) were female and 15.3% (n = 298) were male. In terms of depression, it was observed that the significance varied in the analysis groups for sex (p = 0.039), medication use (p = 0.009) and age (p = 0.010) variables. However, these variables were not significant in some of the subanalysis groups. On the other hand, a p < 0.001 value was obtained for income, physical activity, health perception, body shape perception, life satisfaction, and quality of life variables in terms of depression in the conventional analysis group, and it was seen that the significance continued in all subanalysis groups.

**Conclusions:**

Our findings showed that variables with p < 0.001 in the conventional analysis group maintained their significance in the other analysis groups. In addition, as the p value got closer to 0.05, we observed that the significance changed according to different cutoff points in the analysis groups. In addition, 50% randomly selected samples support these results. At the end of our study, we reached results that support the necessity of secondary tests in the evaluation of scales. Although further studies are needed, we anticipate that our study will shed light on other studies.

## Background

Most phenomena in social and behavioral sciences are not directly assessable, for example, beliefs and emotions, motivational states, and needs; therefore, we develop valid, reliable scales as a measurement tool [1]. For instance, in psychiatry, scales are used for diagnosis along with clinical history and treatment to measure severity and to anticipate or see the response of the disorders. Scales provide standard information because of the items and their function. This allows comparison between different patients or various time points for the same patient. Scales are the appropriate measurement tool to quantify clinical trial outcomes [2].

One of the common psychiatric disorders for which scales are used is depression. Major depressive disorder is a common and serious medical illness that affects the way patients think, feel and behave. It affects an estimated one in 15 adults (6.7%) annually, and one in six (16.6%) will experience depression at some point in their lives. Scales are often used to assess the severity of an illness as difficult to measure as depression [3]. An estimated 3.8% of people suffer from depression, including 5.7% of individuals over the age 60 and 5% of adults (4% of males and 6% of females). Depression affects over 280 million people worldwide [4].

The Beck Depression Inventory was developed for this purpose in 1961 and has been used in academic studies for a long time, and its validity and reliability have been examined in many studies. It has been used by researchers in many countries to rate the severity of depression. As a widely used scale, the BDI has been revised more than once [5–7]. The fact that the participants to whom the scale is applied are distributed in a wide spectrum in terms of sex, age and other sociodemographic characteristics increases its area of use. Many studies have examined the psychometric properties of the scale in samples including the normal population and psychiatric patient groups [8].

A health condition or event can be categorized according to one characteristic or classified based on more than one characteristic. Groups resulting from categorization or classification represent a qualitative definition. The cutoff score is defined as a value or criterion used to mark the lowest point at which a particular status or category is reached [9].

Why do we need to categorize variables? Because of the researchers' belief that it is easier to report and interpret the results, researchers are more familiar with performing studies by categorizing continuous variables, especially because physicians or epidemiologists categorize and use continuous data in their routine practice. For example, the results of blood pressure measurements can be defined as normal, prehypertension, hypertension, etc. [10]. The need for categorization is also seen in scales in daily practice. Due to this need, although cutoff scores are not determined for all scales, cutoff scores are determined specifically for each scale. These cutoff scores help us to divide the scales into at least two or more categories or classes. Since we define quantitative data as qualitative data in this process, they cause various errors.

Categorization errors are one of the reasons for the discrepancy between an unobserved variable and an observed variable that measures it. To give an example of the level of depression, which is our study's latent variable that we cannot directly measure, the level of depression is the unobserved variable, whereas the score obtained with the BDI-II is the observed variable. Specifically, categorization errors are the differences between the score of the latent variable and the observed category resulting from the categorization process.

In the example in our study, a person's depression category could be minimal, mild, moderate or severe. Although no error was made, there may be some inconsistencies between a person's level of depression and the category to which they were assigned. First, people with different scores in certain score ranges are pooled into one category. Second, the distance between categories in terms of average score may not be equal to the unity distances between the numbers assigned to the categories. As we mentioned in the second inconsistency, transformation errors occur when the distances between the numerical scores assigned to each category are not identical distances between the average of the latent variables in each of these categories [11].

Categorization errors occur because infinite possible values of the latent variable are narrowed down to a fixed number of categories. These errors will be higher when there are fewer categories [12].

If the distances between the categories created for the same scale applied in two different countries are not the same, this may lead to greater categorization errors in one country than in the other, leading to lower question quality. However, this difference can also be expected due to differences in data quality and may not be related to categorization error. If category cutoff scores are different across countries, the questions are not directly comparable [12]. Thus, besides random errors and method variance, categorization can be expected to be another source of measurement error [11].

The aim of this study is to evaluate the hypothesis test results after categorizing the scale scores with cut-off points and to assess whether similar results would be obtained in that best represent the categories.

## Methods

### Design

This cross-sectional study was conducted between 15 and 20 March 2023. The survey data were collected via the LimeSurvey platform.

The sample size was calculated with G-power 3.1. The sample size calculated as 1363 with a small effect size (w = 0.10), 0.05 α margin of error, 0.8 power (1-β), the degree of freedom (Df) 6.

The sample was selected by convenience sampling method. Participants were reached via WhatsApp, Facebook, Twitter and Instagram. Survey links were shared on the platforms by the researchers and an Instagram influencer. The eligibility criteria were 18–64 years of age, Turkish citizenship, literacy, and acceptance to participate in the study.

### Ethics

This study was approved by the Ethics Committee of Istanbul University-Cerrahpasa Faculty of Medicine (10.03.2023-638920). Online informed consent was obtained from the all participants. When the survey link was clicked, a page was opened that introduced the study and included an informed consent form. The participants, who clicked on the "I agree to participate in the research" button, reached the page containing the questionnaire and scale. The study was conducted in accordance with the Declaration of Helsinki.

### Measures

The questionnaire consisted of two sections. In the first section, there were questions about the sociodemographic status and life characteristics of the individuals. The Beck Depression Inventory was used in the second section.

### Sociodemographic and life characteristics

The demographic variables were sex and age. Body mass index (BMI) was calculated using self-reported weight and height (kg/m^2^).

The monthly income was evaluated in three categories: “Income less than expenses, equal, higher”.

It is asked about their frequency of reading books: "How often do you read books? (except textbooks or exam books)." The response options were as follows: every day; 1–3 times a week; 1 every 1–2 weeks; 1 per month; 1 in 2–3 months; and not at all. The responses were recoded into three categories: once a week or more (2); 1–3 times a month (1); and once a month or less (0).

According to WHO, at least 150–300 min of moderate intensity or 75–150 min of vigorous intensity or an equivalent combination of Moderate-Vigorous Physical Activity per week is recommended [13]. In this regard, the physical activity was evaluated as sufficient and insufficient physical activity. Insufficient physical activity was determined if the individual did not exercise for at least 60 min twice per week.

Health status was evaluated using a self-rated response. Individuals were asked, “Would you say your health is…?” The response options were excellent, good, fair, and poor. Responses were coded as excellent/good and fair/poor. The chronic disease status asked as 'Do you have a chronic disease that requires medication. The answer options were “no” or “yes” [14].

The Cantril ladder method was used as the life satisfaction measurement. The measure is pictorially presented as a ladder of 11 steps from 0 to 10, where a point of 10 indicates 'best life possible' for the individual and a point of 0 indicates the 'worst possible life.' Individuals were asked, “Where on the ladder do you feel you stand at the moment? [15].

Participants were asked "At what level do you currently feel your quality of life is?" and asked to answer. For quality of life, participants were asked to score between 0 and 100. A score of 100 indicates 'great quality of life', '95 almost great quality of life', '85 very good quality of life', '70 good quality of life', '60 moderately good quality of life', '40 somewhat poor quality of life', '30 poor quality of life', '15 very poor quality of life' and 0 indicates 'extremely poor quality of life'. According to the participants' answers to the Quality of Life question, values of 70 and above were grouped as good quality of life; values of 85 and above were grouped as very good quality of life [16].

To evaluate body shape perception, individuals were asked, “Do you think your body is …..?” Their response options on a 5-point Likert scale ranged from (1) much too thin to (5) much too fat. Responses were recoded into four categories: thin (1 = too thin, and 2 = a bit thin), normal (3 = I have the correct size), a bit fat (4 = a bit fat) and too fat (5 = too fat) [17].

Participants' chronic disease status was assessed with the questions "Do you have a chronic disease that requires medication? If so, what is it? The response options were 'none' and 'yes', with a blank space next to the 'yes' option, and participants who ticked this option were asked to specify their chronic conditions.

### Beck depression inventory II

The Beck Depression Inventory II (BDI-II) was developed by Beck, Steer, Brown in 1996, consists of 21 items and was developed in accordance with DSM-IV criteria for depression. It has high test–retest and internal consistency [18]. The BDI-II contains 21 items on a 4-point scale from 0 (symptom absent) to 3 (severe symptoms). Scoring is achieved by adding the highest ratings for all 21 items. The minimum score is 0, and the maximum score is 63. Higher scores indicate greater symptom severity. Scores of 0–13 indicate minimal depression, 14–19 mild depression, 20–28 moderate depression and 29–63 severe depression [19]. The validity-reliability of the Turkish scale has been demonstrated by different studies. Kapci et al. reported that the internal consistency for the nonclinical and clinical groups was 0.90 and 0.89, respectively; test–retest stability was also high (r = 0.94); and the convergent and discriminant validity results were satisfactory [20]. Uslu et al. reported that the test–retest (r = 0.89) and internal consistency (a = 0.90) reliabilities, convergent validity (r = 0.81) and discriminant validities (r = 0.39, r = 0.49 and r = 0.42) were satisfactory [21].

## Procedures

First, a classical data analysis was conducted with all participants. This group was called the conventional analysis group. Four groups were formed using the cutoff values of the Beck Depression Inventory-II (0–13 for minimal depression, 14–19 for mild depression, 20–28 for moderate depression, 29–63 for severe depression) (Table 1). After the groups were determined, subsamples were formed by taking a certain portion of each group.

**Table 1:** The cutoff values used in the analysis groups for BDI-II

	Minimal depression	Mild depression	Moderate depression	Severe depression
Conventional	0–13	14–19	20–28	29–63
IQR (Q1–Q3)	5–11	15–18	21–25	30–37
± 1 std	4.07–11.49	14.54–17.91	20.55–25.63	29.36–39.11
10% trimmed	1–13	14–19	20–28	29–43
5% trimmed	0–13	14–19	20–28	29–45

**Fig. 1:**
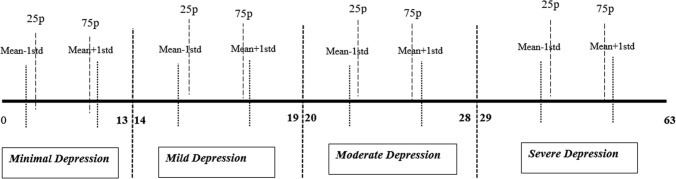
The conventional cut-off values and sub-analysis groups considered to best represent the groups. Only shown for ± 1std and Q1–Q3 for clarity of the figure

We identified 6 subanalysis groups that we think will best represent the groups formed according to the BDI-II. Figure 1 shows the main idea of the study. In the first subanalysis group, those between Q1–Q3 within each BDI-II category were included, and was referred to as IQR group. In the second subanalysis group, a subanalysis group was formed in which those who were within ± 1 std of each BDI-II category were included (± 1std). The third subanalysis group included those 90% of the sample between 5p–95p in each BDI-II category, named as 10% trimmed group. The fourth subanalysis group included 95% of the sample those between 2.5p–97.5p in each BDI-II category, named as 5% trimmed group. In the fifth subanalysis group, 61.8% were included by taking the median value of each BDI-II category as the midpoint (golden ratio). For the third, fourth and fifth and analysis groups, some of the data were excluded from the analysis by simple randomization due to the high number of identical scores at the endpoints. The cutoff values for the other analysis groups are shown in Table 1. Finally, within each BDI-II group, it was randomly selected 100 different samples containing 50% of each group. In addition, their results were given as the proportion of analyses with p < 0.05.

### Statistical analyses

The Statistical Package for the Social Sciences version 21.0 for Windows (IBM Corp., Armonk, NY, USA) was used for data evaluation and analysis. Categorical variables were presented as frequencies (n) and percentages (%), numerical variables were presented as the mean and standard deviation (std) if normally distributed, and median (interquartile range (IQR)) if not normally distributed. The Kolmogorov‒Smirnov test was used to evaluate normality. The chi-square test was used to compare categorical variables. The Kruskal‒Wallis test was used to compare continuous numeric variables between more than two independent samples; adjusted p values were used for post hoc comparisons. The significance level of statistical tests was accepted at p < 0.05.

## Results

**Fig. 2:**
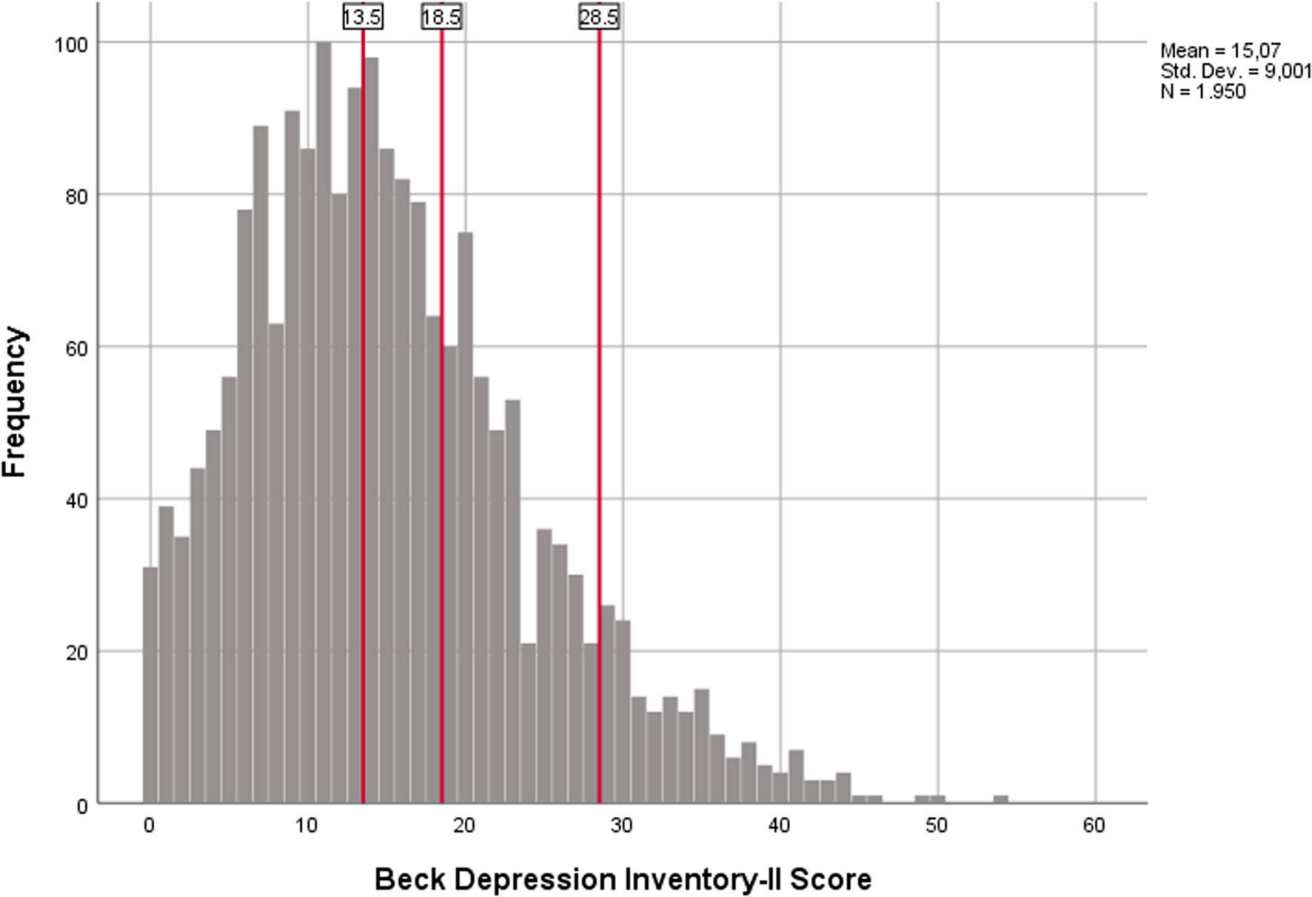
The histogram of Beck Depression Inventory-II scores of participants

Of the 1950 participants, 84.7% (n = 1652) were female and 15.3% (n = 298) were male. Of the participants, 47.9% (n = 935) were in the minimal depression group, 24.1% (n = 469) were in the mild depression group, 19.2% (n = 375) were in the moderate depression group and 8.8% (n = 171) were in the severe depression group. Table 2 shows the number and percentages of people in the depression groups for each analysis group. The histogram of Beck Depression Inventory-II scores of the participants is shown in Fig. 2.

**Table 2:** The frequency of depression categories in the analysis groups

	Minimal depression		Mild depression		Moderate depression		Severe depression	
	n	%	n	%	n	%	n	%
Conventional	935	47.9	469	24.1	375	19.2	171	8.8
IQR (Q1–Q3)	563	47.1	311	26.0	215	18.0	106	8.9
±1std	563	49.2	247	21.6	215	18.8	119	10.4
10% trimmed	841	48.0	423	24.1	337	19.2	151	8.6
5% trimmed	889	48.0	445	24.0	357	19.3	163	8.8
Golden Ratio	551	46.7	290	24.6	233	19.7	106	9.0
Random 50%	468	47.9	235	24.1	188	19.2	86	8.8

Table 3 shows the relationship between depression categories and demographic and socioeconomic life characteristics in the conventional analysis group. Among females, 46.6% (n = 770) had minimal depression, 24.6% (n = 406) had mild depression, 19.6% (n = 324) had moderate depression, and 9.2% (n = 152) had severe depression. Among males, 55.4% (n = 165) had minimal depression, 21.1% (n = 63) had mild depression, 17.1% (n = 51) had moderate depression, and 6.4% (n = 19) had severe depression. There was a significant relationship between sex and depression (p = 0.039). Minimal depression was seen at a lower rate in females than in males. There was no statistically significant relationship between depression and body mass index and reading frequency (p = 0.463, p = 0.901, respectively). There was a statistically significant difference between depression and income level, physical activity, health perception, body shape perception, medication use, life satisfaction and quality of life (p =  < 0.001; < 0.001; < 0.001; 0.001; 0.001; 0.009; < 0.001; < 0.001; < 0.001, respectively).

**Table 3:** The association between depression categories and demographic and socioeconomic life characteristics in the conventional group

	Beck Depression Inventory Scores								p value
	Minimal depression		Mild depression		Moderate depression		Severe depression		
	n	%	n	%	n	%	n	%	
Sex									
Male	165	55.4%	63	21.1%	51	17.1%	19	6.4%	0.039
Female	770	46.6%	406	24.6%	324	19.6%	152	9.2%	
Age (Median; 25.p–75.p)	34 (29–40)		34 (29–39)		33 (28–38)		32 (28–36)		0.010
BMI (Median;25.p–75.p)	23.9 (21.3–26.8)		24 (21.6–27.4)		24.1 (21.5–27.3)		23.9 (20.9–28.1)		0.648
BMI groups									
< 20	120	50.2%	47	19.7%	46	19.2%	26	10.9%	0.463
20–24,99	445	48.3%	224	24.3%	177	19.2%	76	8.2%	
25–29,99	252	48.4%	132	25.3%	99	19.0%	38	7.3%	
> = 30	118	44.0%	66	24.6%	53	19.8%	31	11.6%	
Income									
Income less than expenses	101	31.8%	71	22.3%	95	29.9%	51	16.0%	< 0.001
Income equal to expenses	384	47.3%	198	24.4%	159	19.6%	71	8.7%	
Income higher than expenses	450	54.9%	200	24.4%	121	14.8%	49	6.0%	
Frequency of reading books									
Once a month or less	211	47.8%	109	24.7%	83	18.8%	38	8.6%	0.901
1–3 times a month	284	47.3%	141	23.5%	126	21.0%	49	8.2%	
Once a week or more	440	48.4%	219	24.1%	166	18.3%	84	9.2%	
Physical activity- day/week (Median; 25.p–75.p)	1 (0–3)		1 (0–3)		1 (0–2)		0 (0–2)		< 0.001
Physical activity status									
0–1 day/week	489	43.5%	284	25.2%	240	21.3%	112	10.0%	< 0.001
≥ 2 day/week	446	54.1%	185	22.4%	135	16.4%	59	7.2%	
Self-rated health status									
Fair/poor	309	32.7%	247	26.1%	256	27.1%	134	14.2%	< 0.001
Excellent/good	626	62.4%	222	22.1%	119	11.9%	37	3.7%	
Body shape perception									
Thin	60	37.5%	40	25.0%	42	26.3%	18	11.3%	< 0.001
Normal	504	55.6%	206	22.7%	133	14.7%	63	7.0%	
A bit fat	329	44.2%	183	24.6%	171	23.0%	62	8.3%	
Too fat	42	30.2%	40	28.8%	29	20.9%	28	20.1%	
Usage of medication									
None	689	49.5%	338	24.3%	260	18.7%	105	7.5%	0.009
Yes	246	44.1%	131	23.5%	115	20.6%	66	11.8%	
Life satisfaction									
< 7	348	33.0%	281	26.6%	273	25.9%	153	14.5%	< 0.001
≥ 7	587	65.6%	188	21.0%	102	11.4%	18	2.0%	
Good quality of life (≥ 70)									
Not good quality of life	370	33.6%	292	26.5%	283	25.7%	156	14.2%	< 0.001
Good quality of life	565	66.5%	177	20.8%	92	10.8%	15	1.8%	
Very good quality of life (≥ 85)									
Not very good quality of life	756	43.9%	438	25.4%	361	20.9%	169	9.8%	< 0.001
Very good quality of life	179	79.2%	31	13.7%	14	6.2%	2	0.9%	

There was a statistically significant relationship between age and depression (p = 0.010). The median age of those with minimal depression [34 (29–40)] was significantly higher than the median age of the group with severe depression [32 (28–36)]. There was no statistically significant relationship between BMI and depression (p = 0.648). There was a statistically significant relationship between the frequency of weekly physical activity and depression (p < 0.001). The frequency of weekly physical activity was significantly higher in the minimal depression group than in the other groups.

Table 4 shows the p values obtained in the analyses of all groups. For sex in terms of depression, a statistically significant difference was observed in the conventional analysis group and 5% trimmed groups (p = 0.039; 0.045), but not in the IQR, ± 1std, golden ratio and 10% trimmed groups (p = 0.333; 0.312; 0.309; 0.120). In addition, in 100 random 50% samples, 15% had a p value < 0.05. For 'medication use' in terms of depression, a statistically significant difference was observed in the conventional analysis group and 5% trimmed group (p = 0.009; 0.026) but not in the IQR, ± 1std, golden ratio and 10% trimmed groups (p = 0.645; 0.451; 0.540; 0.072). Also, in 100 random 50% samples, 48% had a p value < 0.05. For 'age' in terms of depression, a statistically significant difference was observed in the conventional analysis group, IQR, ± 1std, golden ratio and 10% trimmed group (p = 0.010; 0.005; 0.014; 0.004; 0.005; 0.005; 0.008). In addition, in 100 random 50% samples, 74% had a p value < 0.05. There was no significant difference in any analyses for BMI or book reading in terms of depression. Also, in 100 random 50% samples, only 2% had a p value < 0.05. In the conventional analysis group, it was statistically significant for income, physical activity, health perception, body shape perception, life satisfaction, and quality of life variables in terms of depression (p < 0.001), the significance persisted in all analysis groups. In addition, in 100 random 50% samples, 100% had a p value < 0.05, for income, self-rated health status, body shape perception, life satisfaction and quality of life. Also, physical activity was found in 97% and physical activity status in 88% with p < 0.05.

**Table 4:** The p values of the comparison of demographic and socioeconomic life characteristics between depression categories in the conventional and all subanalysis groups

Variables	All	IQR	± 1std	Golden Ratio	10% trimmed	5% trimmed	Random %50 samples
	p value	p value	p value	p value	p value	p value	% (p < 0.05)
Sex	**0.039**	0.333	0.312	**0.309**	0.120	**0.045**	**15**
Age	**0.010**	**0.005**	**0.014**	**0.004**	**0.005**	**0.008**	**74**
BMI	0.648	0.786	0.781	0.790	0.669	0.743	**2**
BMI groups	0.463	0.659	0.549	0.688	0.498	0.616	**2**
Income	**< 0.001**	**< 0.001**	**< 0.001**	**< 0.001**	**< 0.001**	**< 0.001**	**100**
Frequency of reading books	0.901	0.731	0.677	0.546	0.980	0.958	**1**
Physical activity	**< 0.001**	**0.005**	**0.003**	**0.010**	**< 0.001**	**< 0.001**	**97**
Physical activity status	**< 0.001**	**0.020**	**0.010**	**0.012**	**< 0.001**	**< 0.001**	**88**
Self- rated health status	**< 0.001**	**< 0.001**	**< 0.001**	**< 0.001**	**< 0.001**	**< 0.001**	**100**
Body shape perception	**< 0.001**	**0.002**	**0.002**	**< 0.001**	**< 0.001**	**< 0.001**	**100**
Usage of medication	**0.009**	0.645	0.451	0.540	0.072	**0.026**	**48**
Life satisfaction	**< 0.001**	**< 0.001**	**< 0.001**	**< 0.001**	**< 0.001**	**< 0.001**	**100**
Good quality of life (≥ 70)	**< 0.001**	**< 0.001**	**< 0.001**	**< 0.001**	**< 0.001**	**< 0.001**	**100**
Very good quality of Life (≥ 85)	**< 0.001**	**< 0.001**	**< 0.001**	**< 0.001**	**< 0.001**	**< 0.001**	**100**

## Discussion

The aim of this study was to evaluate the hypothesis test results after categorizing the scale scores with cut-off points and to assess whether similar results would be obtained in that best represent the categories. This study shows that the variables that were found to be highly significant in our conventional analysis group (i.e., p < 0.01) showed statistical significance again when the tests were repeated in the "IQR", " ± 1std", "golden ratio", "10% trimmed", and "5% trimmed" subanalysis groups. It also had a high percentage of significance in 50% randomly selected samples. At the same time, even if it was significant in the conventional analysis group, the significance varied based on different cutoff points in the subanalysis groups as the p value approached 0.05. The significance of the sex, medication use, and age variables did not remain in all subanalysis groups with regard to depression. The 50% randomly selected samples also had varying but low percentage of significance (gender = 15%, age = 74%, usage of medication = 48%). The p values of income, physical activity, health perception, body shape perception, life satisfaction, and quality of life factors were lower than 0.001 in terms of depression in the conventional analysis group and remained lower than 0.05 in all subanalysis groups. Furthermore, significance remained highly percentage in 50% random samples.

When evaluated in terms of depression, "income" was found to be highly significant in the conventional group analysis (p < 0.001), and it maintained its significance in the subanalysis groups. Many studies support our results regarding the relationship between income and depression. In a study performed by Warden et al., depression was evaluated with 3 different scales. These are the 17-item Hamilton Rating Scale for Depression; 30-item Inventory of Depressive Symptomatology-Clinician-rated; and 16-item Quick Inventory of Depressive Symptomatology-Self-Report. For all three scales, the mean scores that determine the severity of the symptoms decreased from the low- to high-income groups [22]. In a cross-sectional study by Jackson et al., they analyzed African American females. In the study, depression severity and stress were evaluated, and the Beck Depression Inventory II was used for depression assessment as in our study. The results of the study showed that females in the high household income group were less depressed [23].

Many studies have shown the relationship between physical activity and depression. McMahon et al. examined the effects of physical activity on depression in adolescents across multiple countries in the European region and showed that a higher frequency of physical activity was associated with lower levels of depression [24]. In a study conducted by Gorgulu et al., a significant negative correlation was observed between the depression severity score (BDI-II) of those who performed moderate to vigorous physical activity in outpatients. It was also shown that there was an inverse correlation between the severity of depression and physical activity levels in both outpatients and inpatients [25]. As expected, in our conventional analysis group, a significant relationship was found between depression and physical activity (p < 0.001). In our subanalysis groups, the p value continued to maintain significance.

In the conventional analysis group, a statistically significant relationship was found between health status and depression severity. Studies conducted in different sample groups support the results we have obtained. In a cross-sectional study conducted on a primary care population at risk for type 2 diabetes and cardiovascular disease, the health status of participants was assessed by self-rated health (SRH). It is significant that among participants reporting poor or fair physical health, depressive people's perceptions of their physical well-being were lower than those of nondepressive people [26]. In a meta-analysis study examining the relationship between health status and depression in elderly individuals, it was shown that elderly individuals with poor self-rated health had a higher risk (RR: 2.40, 95% CI 1.94–2.97) of depression than those with good self-rated health [27].

In our study, in the conventional analysis group, the percentage of severe depression was significantly higher than that of minimal depression in those who responded as too fat. In the literature, similar findings were found in many studies conducted in different countries [28–32].

In our study, in other analysis groups ("IQR", "1std", "10% trimmed", "5% trimmed", "Golden ratio"), as in the conventional group, a statistically highly significant relationship was found between life satisfaction scoring and depression severity. The fact that similar results were found in studies conducted with different sample groups using different scales in the literature supports our findings. In a study that was carried out in China with 307 participants with no self-reported mental or neurological disorders, it was reported that life satisfaction had a significant negative direct effect on the degree of depression symptoms (β = − 0.301; p = 0.001) [33]. Similarly, negative correlations between life satisfaction and depression have been reported in Poland and Saudi Arabia [34, 35].

As the severity of depression increased, the percentage of those who defined their quality of life as "good" decreased, while the percentage of those who defined their quality of life as "other" increased. Similar results were reported in studies conducted with different sample groups [36, 37].

A significant difference in age distribution among the BDI-II categories was found in the conventional group analysis. Further post hoc analysis revealed that the age of individuals with severe depression significantly lower than that of individuals with minimal depression. Kapci et al. reported that no significant differences were found in sociodemographic characteristics such as age, sex, and educational level in terms of depression [20]. In a cross-sectional study that included 12,376 respondents in Ontario, the highest prevalence of lifetime depression was observed in the 20–24 age group, while the lowest rate was found in the 75 and over age group. Depression prevalence increased with age, peaking at ages 20–24 and gradually declining for participants aged 75 and over [38].

In a cross-sectional study investigating the psychometric properties of the BDI-II in a sample of community-dwelling older and younger adults, there were no significant differences between males and females in mean depression scores. In both younger and older adults, there were no significant differences between the mean scores of males and females [39]. In a cross-sectional study using the BDI-II, among high school athletes, a 2 × 2 ANCOVA with Bonferroni correction for multiple comparisons did not reveal significant differences in total depression symptoms related to age, sex, or their interaction [40]. In our study, in the minimal depression category, the percentage of males was significantly higher than that of females in the conventional group analysis. This significant sex difference in depression categories was consistent in the golden ratio and 5% trimmed groups but not in the other subanalysis groups.

In our study, significant differences in BDI-II categories were observed based on medication usage. While this significance was consistent in the 5% trimmed subanalysis group, it was not maintained in the other subanalysis groups. In a cross-sectional study in a Pakistani population with at least one major chronic disease, predictors of moderate and severe depression included anemia and diabetes. A robust association between depression and chronic diseases, particularly anemia and diabetes, has been highlighted [41].

In this study, the values at the extremes in each group may not reflect the group; therefore, hypothesis tests were conducted on the people who best represented the groups by removing certain people from the extremes. Additionally, there are studies in the literature discussing the differences in the cutoff values of the BDI-II.

The cutoff values suggested by Beck et al. for BDI-II have been analyzed in different countries. The results of these studies support that cutoff scores may vary in different sample groups. In a study conducted in Turkey in 2008 by Runa I Uslu et al. on 669 adolescents, the cutoff value calculated by taking sensitivity, specificity and χ^2^ results into consideration for moderate depression was found to be 4 points lower than the one proposed by Beck et al. in 1996 [21]. In a study by Kathrin Dolle et al. published in 2012 on 88 adolescents aged 13–16 years in Germany, the optimal screening cutoff score was calculated as 23 and above according to the Youden index [42]. In a 2013 systematic review study, it was reported that the BDI-II overestimated the prevalence of depression in sample groups with some particular characteristics; for example, patients with medical illnesses would record more items pertaining to physical complaints. Based on the distinct features of the sample, the optimal cutoff was determined to identify cases of depression syndrome in medical samples. The potential cutoff point varied greatly, ranging from 7 to 22 [43].

The results selectively reported in the literature from only well-performing cutoff scores in diagnostic accuracy studies may bias estimates in meta-analyses [44]. In another study, researchers discovered that the characteristic curves associated with graded statements employing an agree-disagree format generally exhibit relationships with single-peaked, nonmonotonic functions representing actual attitudes. This characteristic tends to maintain greater consistency within an expansion model, such as Thurstone's method, as opposed to the Likert procedure. Moreover, the distinctive response patterns seen in disagree-agree responses can yield inaccurate measures, particularly when individuals with the most extreme attitudes receive Likert scores that suggest more moderate views. Furthermore, it has been reported that the Likert scale's limitations can be exacerbated by constraining the range of attitudes within the sample. Specifically, when the sample's range of attitudes is restricted, the nonmonotonic response characteristic may become obscured, and the characteristic curve may appear to be monotonic in relation to attitude. Consequently, this issue warrants consideration when incorporating items into the Likert scale. If this Likert scale is then applied to a broader array of items within a larger sample, the nonmonotonic characteristics of the statement can resurface, magnifying the validity issues associated with measuring individuals with the most extreme attitudes [45].

When data collected using the Likert scale with more moderate views generate a nonmonotonic, single-peaked curve, we consider the possibility of representing the sample more accurately by including the central portions (or near median) of the curve in our analyses, potentially encompassing the peak, while excluding the extremities of the curve using specified percentages. Taking this representation into consideration, we propose that this approach can serve as a scale sensitivity analysis.

However, in cases where the data exhibit a monotonic curve, there is a tendency for peaks to occur at the curve's extremes. In such instances, utilizing this method as a scale sensitivity analysis may not be advisable, as focusing solely on the middle sections of the curve can diminish the capacity to represent the sample adequately. For datasets characterized by monotonic curves, opting for scales such as the Thurstone-type scale, as opposed to the Likert-type scale, may offer a more precise alternative in terms of scale sensitivity.

## Limitations and strengths

This research is the first study to evaluate that univariate analyses may yield erroneous results in studies using scales with cutoff points and to evaluate the categorization errors of scales that have important usage in social sciences and medicine. In this respect, it is thought that this research will shed light on studies to be conducted in a similar field. This study, which examined the effects of categorization errors and cutoff points on the results of statistical analyses, intended to provide a new perspective on many studies using scales in the literature and to add a new perspective to our view of statistical analyses.

However, this study has some limitations. First, only univariate analyses using scales with cutoff points were affected. We were unable to determine how inaccurate the scales were or how much the use of cutoff scores affected prevalence. Second, we did not take into consideration measurement errors when designing our study. Third, this study was an online survey using convenience sampling and females were more likely to participate. This may have led to fewer males in the sub-analysis groups and may affect the analysis. Also, this leads to a sample with low generalizability to population. Fourth, there was no information in the literature on setting up sub-analysis groups. Therefore, we arbitrarily determined the classical values in biostatistics, namely + 1std, IQR, 90%, 95%, golden ratio. Fifth, sample size may have affected the p value. Further studies are needed to examine the impact of secondary tests on the results of analyses using different scales.

## Conclusions

Our results demonstrated variables that retained their significance in subanalysis groups when they had p < 0.001 in the conventional analysis group. Additionally, we observed that the significance varied based on different cutoff points in the analysis groups as the p value approached 0.05. In addition, 50% randomly selected samples support these results. Especially when the p value obtained as a result of univariate analyses is close to 0.05, it may be appropriate to avoid definite interpretations. In this case, it is among our recommendations to choose a low alpha margin of error (i.e., 0.01) or to proceed to the use of secondary tests. More studies are needed in the literature to evaluate the categorization errors of the scales and the importance of secondary tests on different scales.

## Data Availability

The datasets used and/or analysed during the current study are available from the corresponding author on reasonable request.
